# Resistin is associated with mortality in patients with traumatic brain injury

**DOI:** 10.1186/cc9307

**Published:** 2010-10-28

**Authors:** Xiao-Qiao Dong, Song-Bin Yang, Fang-Long Zhu, Qing-Wei Lv, Guo-Hai Zhang, Hang-Bin Huang

**Affiliations:** 1 Department of Neurosurgery, The First Hangzhou Municipal People's Hospital, 261 Huansha Road, Hangzhou 310000, PR China; 2 Department of Neurosurgery, Shengzhou People's Hospital, 208 Xueyuan Road, Shenzhou 312400, PR China

## Abstract

**Introduction:**

Recently, we reported that high levels of resistin are present in the peripheral blood of patients with intracerebral hemorrhage and are associated with a poor outcome. However, not much is known regarding the change in plasma resistin and its relation with mortality after traumatic brain injury (TBI). Thus, we sought to investigate change in plasma resistin level after TBI and to evaluate its relation with disease outcome.

**Methods:**

Fifty healthy controls and 94 patients with acute severe TBI were included. Plasma samples were obtained on admission and at days 1, 2, 3, 5 and 7 after TBI. Its concentration was measured by enzyme-linked immunosorbent assay.

**Results:**

Twenty-six patients (27.7%) died from TBI within 1 month. After TBI, plasma resistin level in patients increased during the 6-hour period immediately after TBI, peaked within 24 hours, plateaued at day 2, decreased gradually thereafter and was substantially higher than that in healthy controls during the 7-day period. A forward stepwise logistic regression selected plasma resistin level (odds ratio, 1.107; 95% confidence interval, 1.014-1.208; *P *= 0.023) as an independent predictor for 1-month mortality of patients. A multivariate linear regression showed that plasma resistin level was negatively associated with Glasgow Coma Scale score (*t *= -6.567, *P *< 0.001). A receiver operating characteristic curve identified plasma resistin cutoff level (30.8 ng/mL) that predicted 1-month mortality with the optimal sensitivity (84.6%) and specificity (75.0%) values (area under curve, 0.854; 95% confidence interval, 0.766-0.918; *P *< 0.001).

**Conclusions:**

Increased plasma resistin level is found and associated with Glasgow Coma Scale score and mortality after TBI.

## Introduction

Resistin belongs to a novel family of cysteine-rich proteins called resistin-like molecule or found in inflammatory zones (FIZZ) proteins [1]. In rodents, resistin is derived almost exclusively from adipose tissue [2] and implicated as a factor linking obesity and diabetes by impairing insulin sensitivity and glucose tolerance [3]. In humans, resistin is expressed primarily in inflammatory cells, especially macrophages [4]. Furthermore, resistin has been shown to be involved in inflammatory processes. Some proinflammatory agents, such as tumor necrosis factor-α [5], interleukin-6 [6] and lipopolysaccharide [7], can regulate resistin gene expression. Recent studies have shown the regulation of proinflammatory cytokine expression by resistin [8-10]. Moreover, resistin is proposed as an inflammatory marker in human atherosclerosis [11] and rheumatoid arthritis [8].

However, it is evidenced that resistin could be produced by the brain and pituitary gland [12]. Furthermore, resistin mRNA was increased in the cortex of hypoxic and ischemic [13] and traumatic [14] animal brain. In the patients with ischemic stroke, high plasma resistin level has been associated with mortality and disability [15]. Recently, we reported that high levels of resistin are present in the peripheral blood of patients with intracerebral hemorrhage and are associated with poor outcome [16]. However, not much is known regarding change in plasma resistin and its relation to mortality after traumatic brain injury (TBI). Therefore, we examined changes in plasma resistin levels in patients during the initial 7-day period after TBI and also assessed its association with 1-month mortality in a group of TBI patients.

## Materials and methods

### Study population

Between March 2007 and April 2010, a total 119 patients with a postresuscitation Glasgow Coma Scale (GCS) score of 8 or less were admitted to the Department of Neurosurgery, Shengzhou People's Hospital. Exclusion criteria were less than 10 years of age, existing previous neurological disease, head trauma, use of antiplatelet or anticoagulant medication, presence of other prior systemic diseases including uremia, liver cirrhosis, malignancy, chronic heart or lung disease, diabetes mellitus, hyperlipidemia, obesity and hypertension. The patients who suffered severe life-threatening injury to other organs were also excluded. Finally, 94 patients were included.

A control group consisted of 50 healthy age-and sex-matched subjects with normal results on brain magnetic resonance imaging and without vascular risk factors.

Informed consent to participate in the study was obtained from them or their relatives. This protocol was approved by the Ethics Committee before implementation.

### Clinical and radiological assessment

On arrival to the emergency department, a detailed history of vascular risk factors, concomitant medication, GCS score, pupil size and reactivity, body temperature, heart rate, respiratory rate, blood oxygen saturation and blood pressure was taken. Shock was defined as systolic blood pressure less than 90 mmHg [17]. Hypoxia was defined as blood oxygen saturation less than 85% [17]. Hyperglycemia was defined as blood glucose more than 11.1 mmol/L [18]. Hypoglycemia was defined as blood glucose less than 2.2 mmol/L [19]. Neurology deterioration was defined as occurring in patients who manifested clinically identified episodes of one or more of the following: (1) a spontaneous decrease in GCS motor score of 2 points or more from the previous examination, (2) a further loss of papillary reactivity, (3) development of papillary asymmetry greater than 1 mm, or (4) deterioration in neurological status sufficient to warrant immediate medical or surgical intervention [17].

All computed tomography (CT) scans were performed according to the neuroradiology department protocol. Investigators who read them were blinded to clinical information. Focal mass lesion, midline shift > 5 mm, abnormal basal cisterns (compressed or absent cisterns) and traumatic subarachnoid hemorrhage were recorded. Focal mass lesions included contusion, subdural hematoma, epidural hematoma and intracerebral hematoma. CT classification was performed using Traumatic Coma Data Bank criteria on initial postresuscitation CT scan according to the method of Marshall *et al*. [20].

### Patient management

The treatments included surgical therapy, ventilatory support, arterial pressure maintenance, glycemic control, intravenous fluids, hyperosmolar agents, H_2 _blockers, early nutritional support and physical therapy. The decision to intubate and use mechanical ventilation was based on the individuals' level of consciousness, ability to protect their airway and arterial blood gas levels [21]. Adequate intravascular volume was pursued aggressively, and vasopressors were used only after volume expansion. When clinical and radiological examinations provided an estimate of elevation of intracranial pressure, osmotherapy in the form of intravenous mannitol was administered, if available, deepening sedation and hyperventilation. Hyperglycemia and hypoglycemia were strictly avoided. Intracranial mass lesions associated with midline displacement greater than 5 mm were surgically removed when necessary. If intracranial pressure remained high despite maximal medical therapy or after intracranial mass lesion was removed, decompressive craniectomy was performed as soon as possible.

### Determination of resistin in plasma

The informed consents were obtained from the study population or family members in all cases before the blood was collected. In the control group, venous blood was drawn at study entry. In the TBI patients, venous blood was drawn on admission (defined as day 0) and at 8:00 AM at days 1, 2, 3, 5 and 7 after TBI. The blood samples were immediately placed into sterile ethylenediaminetetraacetic acid test tubes and centrifuged at 1500 *g *for 20 minutes at 4°C to collect plasma. Plasma was stored at -70°C until assayed. The concentration of resistin in plasma was analyzed by enzyme-linked immunosorbent assay using commercial kits (R&D Systems, Minneapolis, MN, USA) in accordance with the manufacturer's instructions.

### End point

Outcome was assessed as mortality within 1 month. Cause of death during the study for all patients was TBI.

### Statistical analysis

All values are expressed as means ± standard deviation (SD) unless otherwise specified. Statistical analysis was performed with SPSS 10.0 software (SPSS Inc., Chicago, IL, USA) and MedCalc 9.6.4.0 software (MedCalc Software, Mariakerke, Belgium), and included the Mann-Whitney *U *test, χ^2 ^test, Fisher's exact test, Spearman correlation coefficient, *z *statistic analysis, forward stepwise logistic regression and multivariate linear regression. A receiver operating characteristic curve was configured to establish the cutoff point of plasma resistin with the optimal sensitivity and specificity for predicting 1-month mortality. A *P *value less than 0.05 was considered statistically significant.

## Results

### Patient characteristics

Ninety-four patients were enrolled in this study, namely, 67 men and 27 women. The mean age was 42.9 ± 18.6 years (range, 11-80 years). On admission, the mean GCS score was 5.8 ± 1.8 (range, 3-8), the mean systolic arterial pressure was 123.5 ± 29.5 mmHg (range, 50-180 mmHg), the mean diastolic arterial pressure was 73.9 ± 18.9 mmHg (range, 30-108 mmHg) and the mean arterial pressure was 90.4 ± 21.5 mmHg (range, 38.0-124.7 mmHg). Fourteen (14.9%) patients suffered from shock, 18 (19.1%) patients had hyperglycemia, 3 (3.2%) patients had hypoglycemia, 9 (9.6%) patients had hypoxia and 38 (40.4%) patients had unreactive pupils. Seventy-eight patients (83.0%) need mechanical ventilation. On initial CT scan, 34 (36.2%) patients had abnormal cisterns, 40 (42.6%) patients showed midline shift > 5 mm and 48 (51.1%) patients had the presence of traumatic subarachnoid hemorrhage. After admission, 21 (22.3%) patients presented with neurological deterioration. In the first 24 hours, 40 (42.6%) patients underwent intracranial surgery. Forty-five (47.9%) patients had CT classification of 5 or 6. The mean admission time was 2.2 ± 1.4 hours (range, 0.5-8.0 hours) after TBI. The mean plasma-sampling time was 3.0 ± 1.4 hours (range, 1.0-8.4 hours) after TBI. The baseline blood glucose level was 9.3 ± 3.2 mmol/L (range, 1.4-17.6 mmol/L). The baseline plasma C-reactive protein, fibrinogen, D-dimer and resistin levels were 7.7 ± 2.6 mg/L (range, 3.9-13.4 mg/L), 4.1 ± 2.0 g/L (range, 1.6-8.3 g/L), 2.2 ± 1.0 mg/L (range, 1.0-4.9 mg/L) and 28.1 ± 12.2 ng/mL (range, 10.2-69.7 ng/mL), respectively.

### Serial change in plasma resistin level in patients with TBI

**Figure 1:**
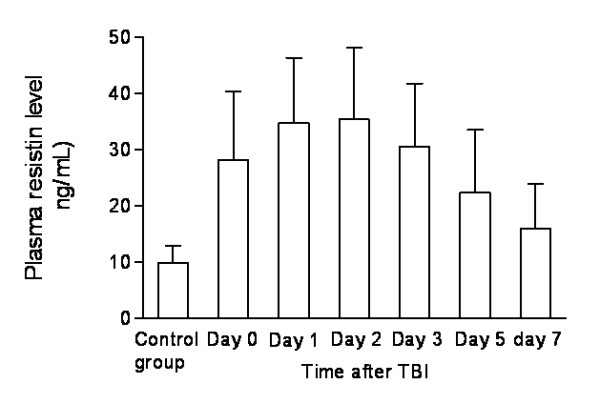
**Graph showing serial changes of plasma resistin concentration in traumatic brain injury (TBI) patients**. Data are expressed as means ± SD.

After TBI, plasma resistin level in patients increased during the 6-hour period immediately, peaked within 24 hours, plateaued at day 2, decreased gradually thereafter and was substantially higher than that in healthy controls during the 7-day period (Figure 1).

### Mortality prediction

Twenty-six patients (27.7%) died from TBI within 1 month. Baseline plasma resistin level in the nonsurvival group was significantly higher than that in the survival group (39.4 ± 12.4 vs. 23.8 ± 9.0 ng/mL; *P *< 0.001). The neurological condition upon admission using GCS score and unreactive pupils was statistically significantly different (both *P *< 0.001) between the two groups. A higher proportion of patients in the nonsurvival group suffered from hyperglycemia (*P *= 0.003), had CT classification of 5 or 6 (*P *= 0.036) and required mechanical ventilation (*P *= 0.007) compared with those in the survival group. The brain CT scan results on admission were analyzed and demonstrated a statistically significant difference between the two groups in abnormal cisterns (*P *< 0.001), in midline shift > 5 mm (*P *= 0.001) and in the presence of traumatic subarachnoid hemorrhage (*P *= 0.029). Blood glucose level (*P *= 0.038) and plasma C-reactive protein (*P *= 0.007), fibrinogen (*P *= 0.015) and D-dimer (*P *= 0.011) levels in the survival group were significantly lower than those in the nonsurvival group in the laboratory examination results on admission.

When the above variables found to be significant in the univariate analysis were introduced into the logistic model, multivariate analyses selected GCS (odds ratio, 0.294; 95% confidence interval, 0.153-0.565; *P *< 0.001) and plasma resistin level (odds ratio, 1.107; 95% confidence interval, 1.014-1.208; *P *= 0.023) as the independent predictors for 1-month mortality of patients.

### Correlations of plasma resistin level with GCS scores

A significant correlation emerged between GCS score and plasma resistin level, as well as other variables shown in Table 1. When the above variables were introduced into the linear regression model, plasma resistin level remained negatively associated with GCS score (*t *= -6.567; *P *< 0.001).

**Table 1:** Baseline clinical, radiological and laboratory factors correlated with plasma resistin level*

	*r *value	*P *value
GCS score on admission	-0.547	0.000
Hyperglycemia on admission	0.333	0.001
Hypoxia on admission	0.286	0.005
Pupils unreactive on admission	0.521	0.000
CT classification 5 or 6	0.219	0.034
Abnormal cisterns on initial CT scan	0.344	0.001
Midline shift > 5 mm on initial CT scan	0.308	0.002
Mechanical ventilation	0.223	0.031
Blood glucose level (mmol/L)	0.241	0.019
Plasma C-reactive protein level (mg/L)	0.332	0.001
Plasma fibrinogen level (g/L)	0.281	0.006
Plasma D-dimer level (mg/L)	0.232	0.025

*Correlations of plasma resistin level with other variables were analyzed by Spearman test. CT, computed tomography; GCS, Glasgow Coma Scale.

### The predictive significance of plasma resistin level for 1-month mortality of patients

**Figure 2:**
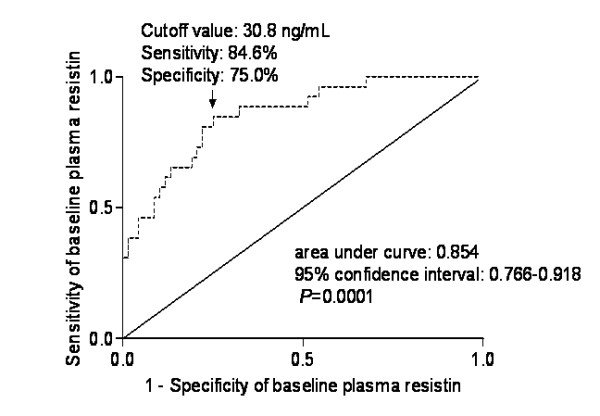
**Graph showing the predictive significance of plasma resistin level for 1-month mortality of patients**. Receiver operating characteristic curve was analyzed by *z *statistic analysis.

A receiver operating characteristic curve identified that a plasma resistin level predicted 1-month mortality of TBI patients with optimal sensitivity and specificity (Figure 2).

## Discussion

Resistin is generally considered to be exclusively produced by adipose tissue [1]. Nevertheless, there is little doubt that the resistin gene is expressed in multiple nonadipose sites. Resistin expression is abundant in the brain and pituitary gland [12]. This abundance of nonadipose tissue sites for resistin expression has complicated the original hypothesis that resistin might be an important link between adipocytes and insulin resistance. Recently, it was evidenced that resistin mRNA was increased in the cortex of hypoxic/ischemic [13] and traumatic [14] animal brain. This protein also increases in the peripheral blood of patients with ischemic [15] and hemorrhagic [16] stroke. These findings suggest that resistin could contribute to the pathogenesis of brain injury.

This study found increased plasma resistin level after acute (< 6 hours) severe TBI in association with a worse clinical outcome. It is well known that high plasma resistin levels may be strongly associated with an increased risk of 5-year mortality or disability after atherothrombotic ischemic stroke [15] as well as related to 1-week mortality after acute spontaneous basal ganglia hemorrhage [16]. To our knowledge, this is the first time that the relationship of plasma resistin level with outcome has been investigated soon after TBI in adults. In this study, a low score on the GCS upon admission was strongly correlated with a high plasma resistin level. A multivariate analysis selected plasma resistin level as an independent predictor of mortality. Overall, it was suggested that plasma resistin level in this early period might reflect the initial brain injury.

The present work is a single-institution study and has the inherent limitations of any small series. As a consequence, the conclusions in this study remain to be verified.

## Conclusions

In this study, increased plasma resistin level is found and associated with GCS score and mortality after TBI.

## Key messages

• In patients with traumatic brain injury, plasma resistin level increased during the 6-hour period immediately, peaked within 24 hours, plateaued at day 2, decreased gradually thereafter and was substantially higher than that in healthy controls during the 7-day period.

• Plasma resistin levels were highly associated with GCS scores after traumatic brain injury.

• Resistin could possibly serve as a novel biomarker in TBI.

• Plasma resistin level predicted 1-month mortality after TBI with the high sensitivity and specificity values.

• Resistin may be a good prognostic factor for mortality in patients with TBI.

## Abbreviations

CT: computed tomography; GCS: Glasgow Coma Scale; TBI: traumatic brain injury.

## Competing interests

The authors declare that they have no competing interests.

## Authors' contributions

XQD, SBY and FLZ contributed to the design of the study, drafted the manuscript and participated in the laboratory work. SBY, QWL, GHZ and HBH enrolled the patients. XQD and SBY contributed to data analysis and interpretation of the results. All authors read and approved the final manuscript.
